# Timing-dependent renal protection of dapagliflozin in endotoxemic diabetic mice by real-time GFR and biomarkers

**DOI:** 10.1186/s40635-026-00940-2

**Published:** 2026-07-01

**Authors:** Yu Xiao, Lifeng Shen, Yuchen Song, Yanqi Liu, Yu Xin, Yinghao Luo, Qianqian Zhang, Weiting Zhang, Xinran Wang, Xiuhua Zhang, Xibo Wang, Pengfei Huang, Fengye Liu, Xianglin Meng, Kaijiang Yu, Changsong Wang

**Affiliations:** Department of Critical Care Medicine, First Affiliated Hospital of Harbin Medical University, Harbin, 150001 Heilongjiang China

**Keywords:** Acute kidney injury, Endotoxemia, SGLT2 inhibitors, Diabetes mellitus, Glomerular filtration rate, Lipopolysaccharide, Diabetic kidney disease, AKI and critical care

## Abstract

**Introduction:**

Type 2 diabetes mellitus (T2DM) increases susceptibility to sepsis-associated acute kidney injury (SA-AKI). While guidelines support chronic sodium-glucose cotransporter 2 (SGLT2) inhibitor use in T2DM, their efficacy and optimal intervention timing in acute sepsis remain unclear. We hypothesized that this efficacy is timing-dependent. To test this, we utilized a T2DM mouse model subjected to graded lipopolysaccharide (LPS) challenges as a clinically relevant surrogate for sepsis. Notably, to precisely capture the longitudinal and dynamic trajectory of renal function, we incorporated continuous real-time transcutaneous glomerular filtration rate (tGFR) monitoring. We evaluated the SGLT2 inhibitor dapagliflozin (DAPA) across three regimens representing distinct clinical scenarios: preventive (pre-LPS only, modeling prior chronic use), therapeutic (initiated post-LPS, modeling de novo intensive care initiation), and whole-course (continuous administration, modeling treatment continuation).

**Results:**

Following LPS challenge, T2DM exacerbated renal dysfunction across all doses and impaired endogenous functional recovery. High-resolution real-time tGFR tracking and subsequent biomarker analyses revealed that the renoprotective effect of DAPA was dependent on the timing of intervention. In moderate endotoxemia, both therapeutic and whole-course regimens were superior to the preventive regimen. Compared to preventive administration alone, initiating or continuing treatment during the acute insult (therapeutic and whole-course regimens) more effectively preserved early glomerular filtration rate (GFR) trajectories, attenuated the rise in tubular injury markers, and promoted histological repair.

**Conclusions:**

Supported by dynamic renal function monitoring, this study demonstrates that DAPA provides significant renoprotection in a preclinical T2DM model of endotoxemia-associated acute kidney injury. This efficacy is closely associated with the timing of intervention, with greater protective effects observed when treatment is initiated during or maintained after the acute endotoxemic challenge. These preclinical findings suggest that intervention timing could be a key determinant of SGLT2 inhibitor efficacy, providing a scientific rationale for evaluating the prompt initiation or continuation of this therapy in diabetic patients presenting with acute sepsis.

**Supplementary Information:**

The online version contains supplementary material available at 10.1186/s40635-026-00940-2.

## Introduction

Patients with type 2 diabetes mellitus (T2DM) face a substantially higher incidence of sepsis-associated acute kidney injury (SA-AKI). A large‑scale study of 12.5 million sepsis patients revealed a significantly higher acute kidney injury (AKI) incidence in diabetics (13% vs. 7%) [1], and subsequent meta-analyses confirmed that T2DM increases this risk by over 50% [2, 3]. Despite this high risk, specific evidence-based organ support strategies for this population remain limited.

Identifying effective renoprotective strategies for T2DM patients is therefore imperative. Sodium–glucose cotransporter 2 (SGLT2) inhibitors have emerged as promising agents due to their demonstrated cardiovascular and renal benefits, leading the American Diabetes Association (ADA) to strongly recommend them to mitigate complications in T2DM [4, 5].

However, guideline recommendations are primarily based on chronic, stable conditions, and guidance for acute critical illnesses like sepsis remains limited [5]. Consequently, existing clinical evidence regarding the renoprotective effect of SGLT2 inhibitors in SA-AKI is strikingly inconsistent. On one hand, large-scale meta-analyses and observational studies suggest that SGLT2 inhibitor use is associated with a markedly reduced risk of sepsis and AKI in chronic settings [6–11]. On the other hand, a 2023 prospective pilot study conducted in a critical care setting—where over half the patients had sepsis—found no significant renoprotective effect of empagliflozin [12]. This stark contradiction suggests that well-documented chronic benefits may not directly translate to the acute, dynamic context of sepsis, leaving a critical knowledge gap.

We hypothesize that a fundamental reason for this efficacy gap lies in how SGLT2 inhibitors interact with distinctly different pathophysiological states. In chronic diabetic nephropathy, SGLT2 inhibitors provide sustained renoprotection by gradually attenuating established hemodynamic and metabolic dysregulation. In contrast, SA-AKI is a rapidly evolving inflammatory storm and acute cellular energetic crisis. While the core mechanisms of SGLT2 inhibitors—such as activating tubuloglomerular feedback (TGF), preserving cellular energetics, and mitigating oxidative stress [13]—theoretically target both conditions, their functional role likely shifts depending on the timing of administration. Consequently, preventive administration may merely modulate pre-insult homeostasis, whereas initiating treatment synchronously with the acute injury peak could function as an active interventional rescue, effectively intercepting the severe metabolic crisis. No studies have yet systematically explored this therapeutic time window.

To directly test this timing hypothesis, we designed a preclinical study utilizing a combined murine model of T2DM subjected to graded LPS-induced endotoxemia. Although LPS primarily induces sterile endotoxemia, recent transcriptomic analyses demonstrate it shares profound early-phase molecular similarities with gold-standard polymicrobial models [14]. Thus, this graded approach serves as a robust, translationally relevant surrogate to simulate varying clinical sepsis severities. To capture the dynamic trajectory of acute renal injury, we employed continuous real-time transcutaneous glomerular filtration rate (tGFR) monitoring. This high-resolution approach enabled us to evaluate dapagliflozin (DAPA) across a multi-time-window protocol: preventive (pre-insult), therapeutic (post-insult), or whole-course administration. Crucially, this design directly mirrors real-world clinical scenarios. Specifically, the preventive arm models patients on prior guideline-directed therapy, the therapeutic arm mimics de novo initiation in the intensive care unit (ICU), and the whole-course arm represents continuing pre-existing therapy during acute sepsis. Ultimately, this study aims to clarify whether renal protection is best achieved via pre-conditioning, acute-phase interception, or sustained management, thereby providing time-resolved evidence to optimize SGLT2 inhibitor use in critically ill diabetic patients.

## Results

### T2DM exacerbates endotoxemia-induced renal dysfunction in a dose- and time-dependent manner

**Fig. 1:**
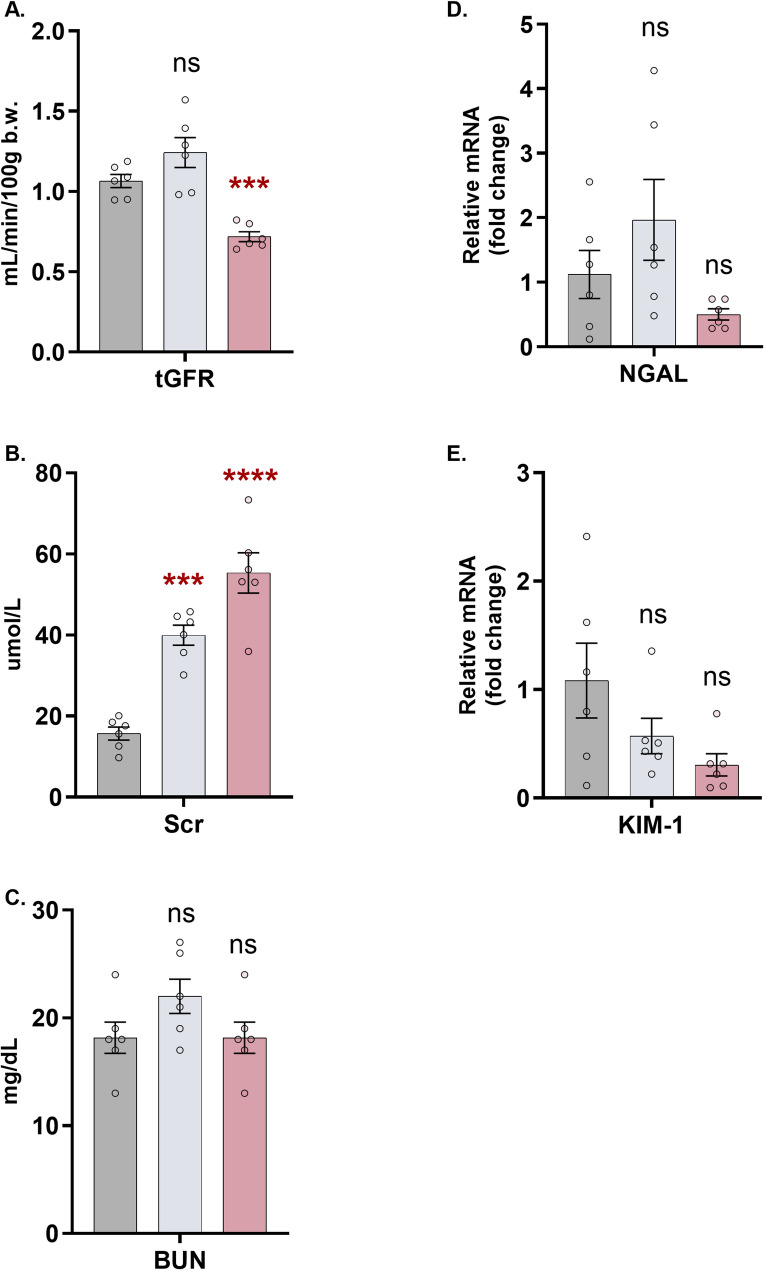
Baseline renal function and injury markers. Baseline parameters were measured in WT control mice (dark gray), WT mice receiving 7-day preventive DAPA (light gray), and untreated T2DM mice (pink). The panels display (**A**) tGFR, (**B**) Scr, (**C**) BUN, and the relative renal mRNA expression (fold change) of (**D**) *NGAL* and (**E**) *KIM-1*. Red asterisks and “ns” above the bars indicate the level of statistical significance compared to the WT control group (*P < 0.05, ***P < 0.001, ****P < 0.0001; ns, not significant). Data are expressed as mean ± SEM. b.w., body weight; BUN, blood urea nitrogen; DAPA, dapagliflozin; KIM-1, kidney injury molecule-1; NGAL, neutrophil gelatinase-associated lipocalin; Scr, serum creatinine; T2DM, type 2 diabetes mellitus; tGFR, transcutaneous glomerular filtration rate; WT, wild-type

At baseline, T2DM mice exhibited a lower glomerular filtration rate (GFR) compared to wild-type (WT) controls. Regarding serum creatinine (Scr), levels in WT mice receiving DAPA were higher than those in untreated controls but remained within the normal murine reference range (17.7–70.7 μmol/L). Conversely, the untreated T2DM group showed elevated Scr levels, with one animal slightly exceeding the upper limit of this range. Despite these functional variations, the renal injury biomarkers neutrophil gelatinase-associated lipocalin (NGAL) and kidney injury molecule-1 (KIM-1) remained comparable across all groups, and DAPA administration did not adversely affect these structural markers in WT mice (Fig. 1).

**Fig. 2:**
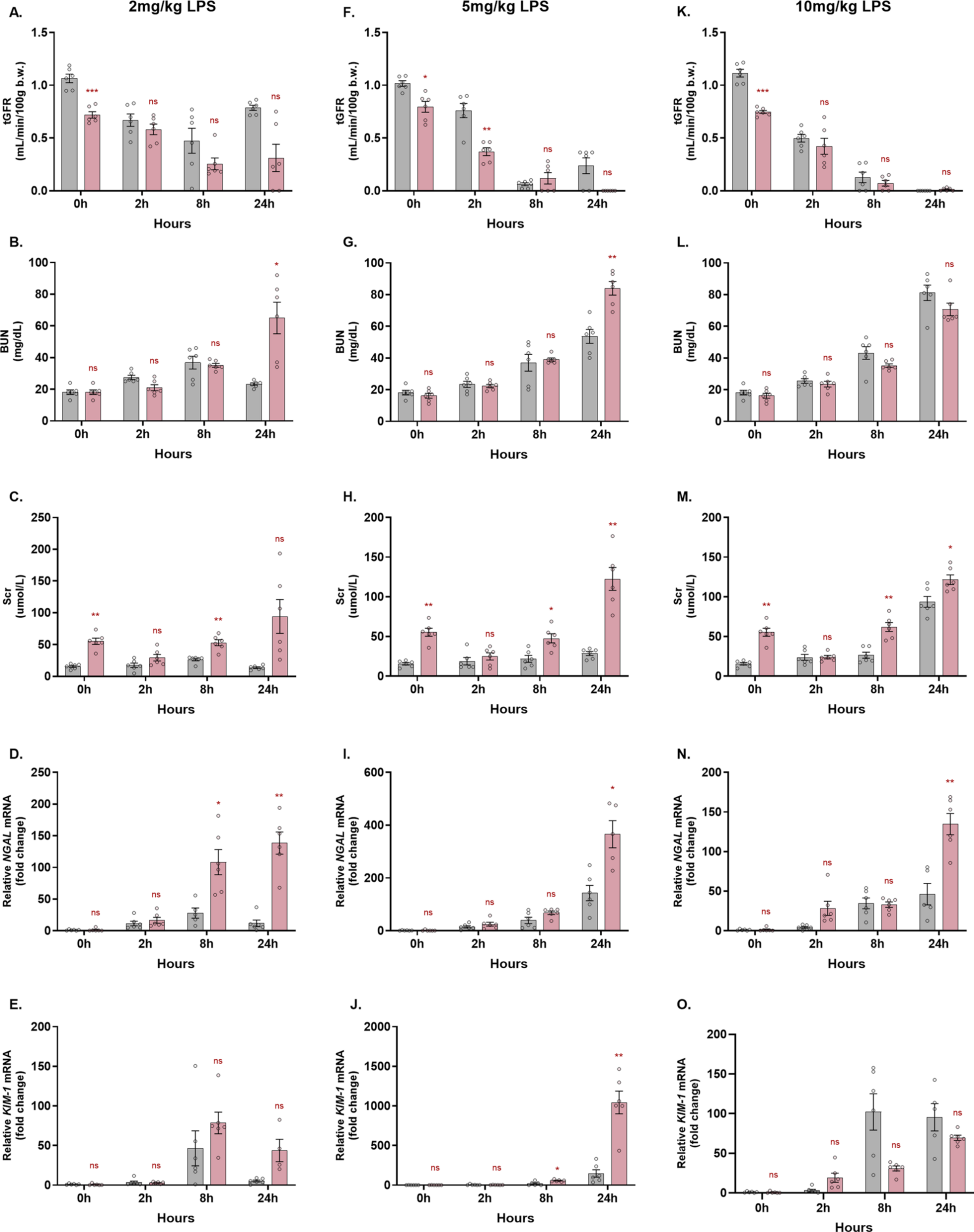
Renal function and injury markers following LPS challenge in WT and T2DM mice. Parameters were measured in WT (dark gray) and T2DM (pink) mice at 0, 2, 8, and 24 h following i.p. injection of LPS at 2 mg/kg (**A**–**E**), 5 mg/kg (**F**–**J**), or 10 mg/kg (**K**–**O**). The panel displays tGFR (**A**, **F**, **K**), BUN (**B**, **G**, **L**), Scr (**C**, **H**, **M**), and the renal mRNA expression of *NGAL* (**D**, **I**, **N**) and *KIM-1* (**E**, **J**, **O**). Red asterisks and “ns” above the bars indicate the level of statistical significance between the WT and T2DM groups at the corresponding time point and dose (*P < 0.05, **P < 0.01, ***P < 0.001; ns, not significant). Data are expressed as mean ± SEM. b.w., body weight; BUN, blood urea nitrogen; h, hours; i.p., intraperitoneal; KIM-1, kidney injury molecule-1; LPS, lipopolysaccharide; NGAL, neutrophil gelatinase-associated lipocalin; Scr, serum creatinine; T2DM, type 2 diabetes mellitus; tGFR, transcutaneous glomerular filtration rate; WT, wild-type

Following lipopolysaccharide (LPS) challenge, renal function declined in a dose- and time-dependent manner in both control and T2DM mice. However, this deterioration was more pronounced in the T2DM group. Compared to controls, T2DM mice exhibited a greater reduction in GFR and higher Scr and BUN levels at each LPS dose and time point. Furthermore, the progression of endotoxemia-induced injury over 24 h was more rapid, and functional recovery was impaired in T2DM mice (Fig. 2).

### The renoprotective effect of dapagliflozin in endotoxemia is timing-dependent: therapeutic and whole-course regimens outperform preventive administration

**Fig. 3:**
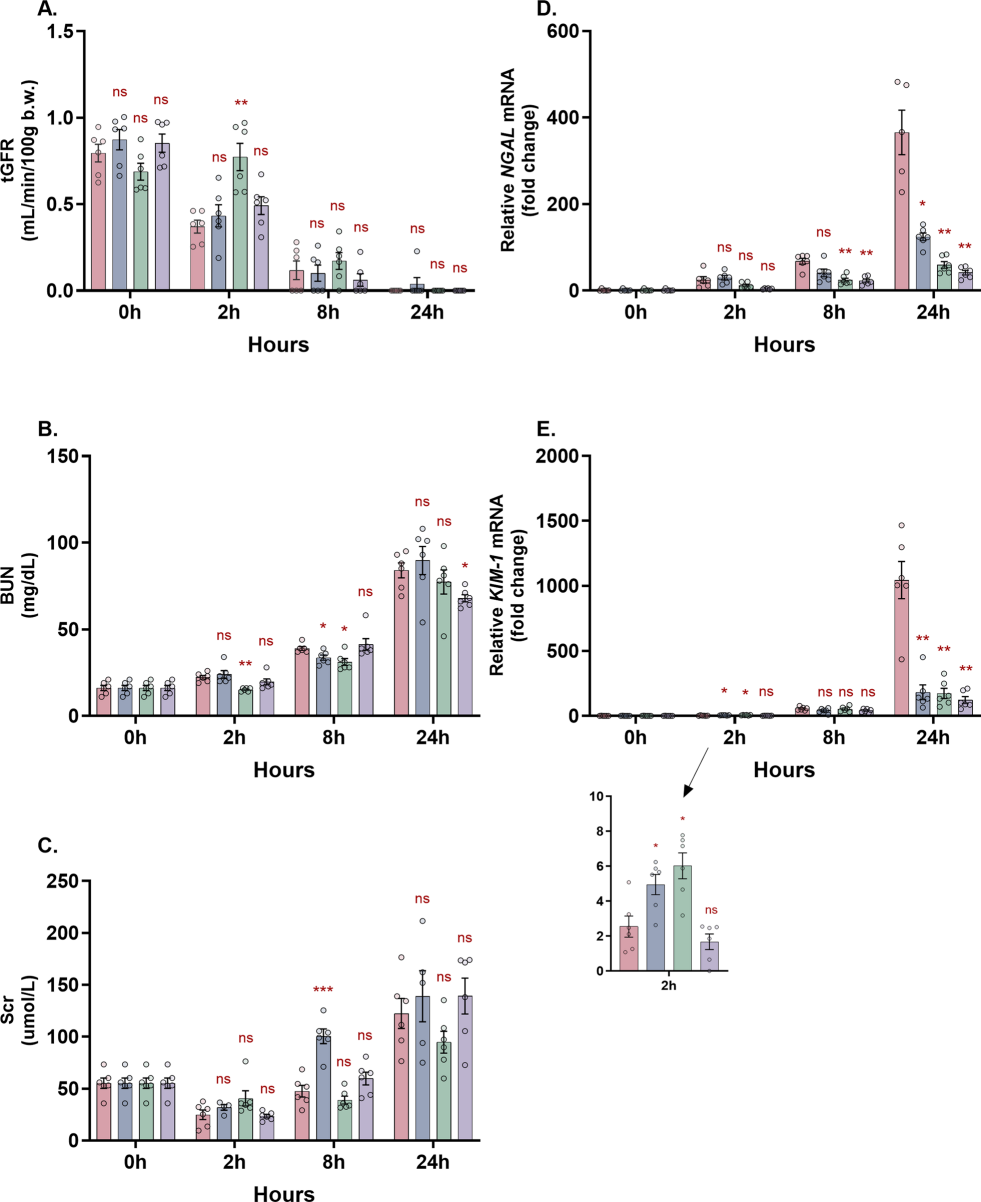
Renal function and injury markers across DAPA regimens in T2DM mice following moderate-dose LPS challenge. Parameters were measured in T2DM mice at 0, 2, 8, and 24 h following i.p. injection of 5 mg/kg LPS under four regimens: untreated (pink), Preventive DAPA (blue), Therapeutic DAPA (green), and Whole-course DAPA (purple). The panel displays (**A**) tGFR, (**B**) BUN, (**C**) Scr, and the renal mRNA expression of (**D**) *NGAL* and (**E**) *KIM-1*. Red asterisks and “ns” above the bars indicate the level of statistical significance compared to the untreated T2DM group at the corresponding time point (*P < 0.05, **P < 0.01, ***P < 0.001; ns, not significant). Data are expressed as mean ± SEM. b.w., body weight; BUN, blood urea nitrogen; DAPA, dapagliflozin; h, hours; i.p., intraperitoneal; KIM-1, kidney injury molecule-1; LPS, lipopolysaccharide; NGAL, neutrophil gelatinase-associated lipocalin; Scr, serum creatinine; T2DM, type 2 diabetes mellitus; tGFR, transcutaneous glomerular filtration rate

Dynamic GFR monitoring and biomarker analysis revealed distinct, time-dependent patterns of renal protection across the three DAPA regimens (Fig. 3).

At 2 h post-LPS, GFR was already significantly decreased in the T2DM + LPS (disease) group. At this early injury phase, only the therapeutic regimen (initiated after LPS) demonstrated a significant protective effect, attenuating the decline in GFR compared to the disease group (*P* < 0.05). In contrast, both the preventive and whole-course regimens showed GFR values that were not statistically different from the disease group, indicating a lack of immediate early benefit on glomerular filtration from drug exposure prior to the endotoxemic insult.

By 8 h post-LPS, progressive injury caused further GFR declines across all groups, eliminating inter-group statistical differences. However, analysis of tubular injury markers revealed emerging patterns: the therapeutic regimen showed a dominant trend in attenuating the rise of *NGAL* and *KIM-1* mRNA levels compared to the preventive regimen.

At 24 h post-LPS, the timing-dependent effects became more pronounced across different readouts. The "therapeutic regimen" provided significantly greater suppression of the tubular injury markers NGAL and KIM-1 compared to the preventive regimen (*P* < 0.05). For the conventional azotemia indicators (BUN and Scr), the protective effects of the preventive and therapeutic regimens were similar in magnitude. Strikingly, the "whole-course regimen" consistently demonstrated the most comprehensive protection, showing the smallest magnitude of deterioration across the entire time series for multiple indicators (GFR trend, BUN, Scr, NGAL, and KIM-1). This pattern highlights the cumulative advantage of continuous drug coverage before, during, and after the endotoxemic challenge.

Collectively, these data indicate a timing-dependent effect of DAPA. Preventive administration showed limited benefit in early functional preservation, whereas the therapeutic regimen attenuated the initial GFR decline and reduced tubular injury markers. Furthermore, the whole-course regimen yielded the most consistent improvement across all evaluated parameters, suggesting the advantage of sustained pharmacological coverage.

### Histopathological validation of structural recovery during endotoxemia

**Fig. 4:**
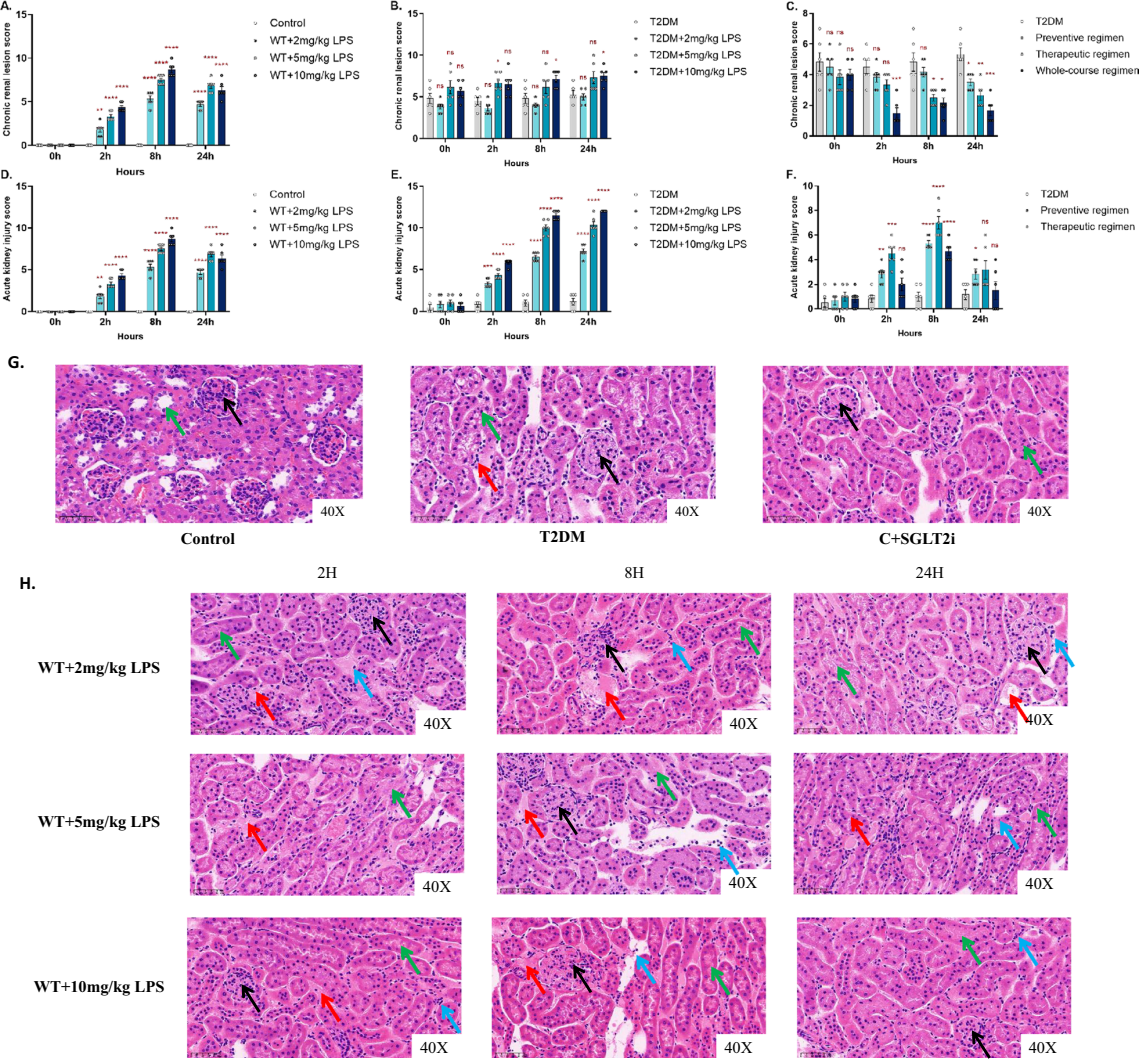
Renal injury scores across all groups, and representative pathology of baseline and LPS-challenged WT mice. Panels (**A**–**F**) display the acute and chronic semi-quantitative histopathological injury scores encompassing all experimental groups in the study. Panels (**G**) and (**H**) present representative H&E-stained kidney sections (scale bar = 50 μm) for the baseline groups (WT control, untreated T2DM, and WT receiving preventive DAPA), as well as WT mice evaluated at 2, 8, and 24 h following i.p. injection of 2, 5, or 10 mg/kg LPS, respectively. Asterisks indicate the level of statistical significance (*P < 0.05, **P < 0.01, ***P < 0.001). Data are expressed as mean ± SEM. DAPA, dapagliflozin; h, hours; H&E, hematoxylin and eosin; i.p., intraperitoneal; LPS, lipopolysaccharide; T2DM, type 2 diabetes mellitus; WT, wild-type

**Fig. 5:**
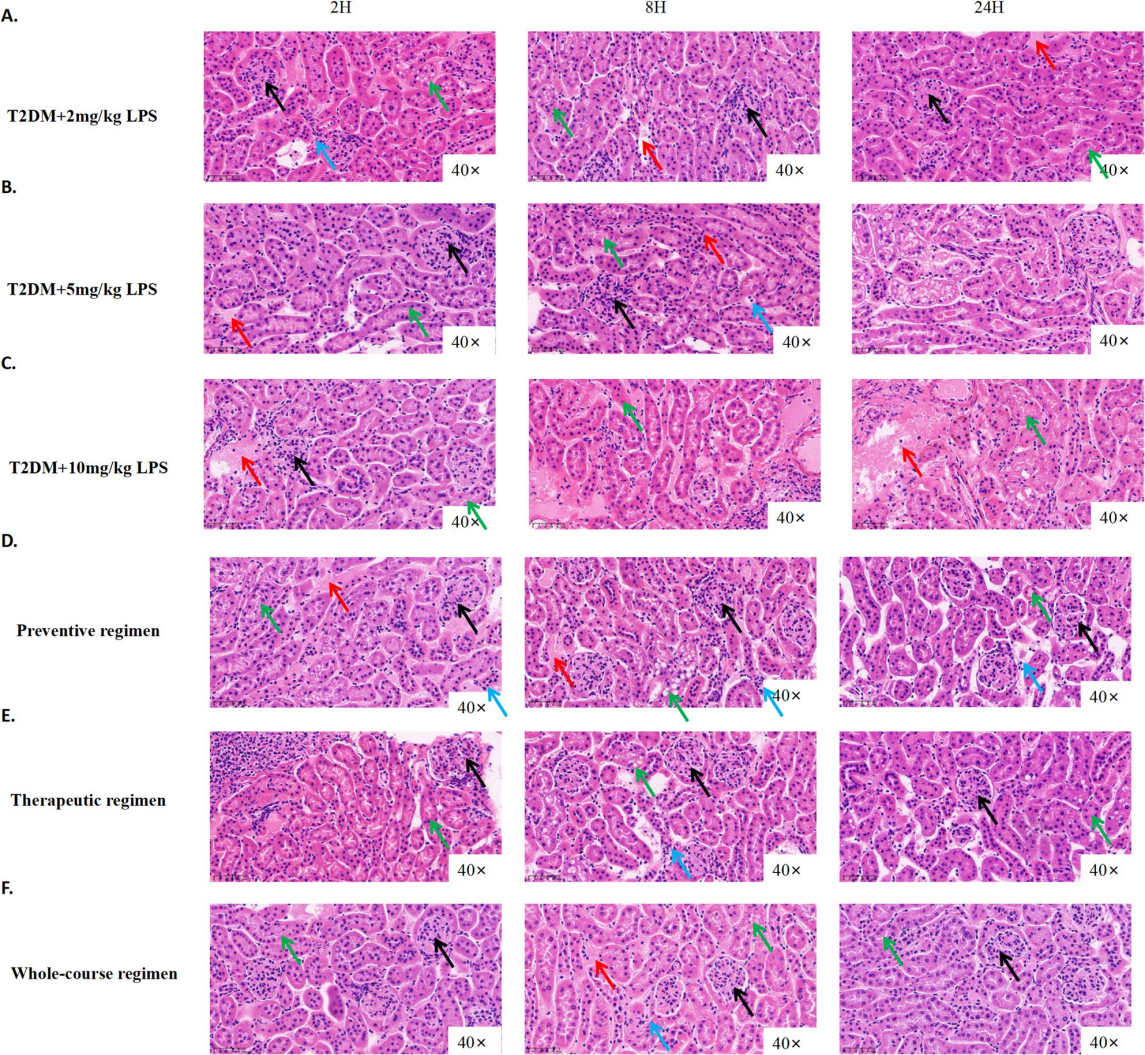
Renal histopathology in LPS-challenged T2DM mice and the effects of different DAPA regimens. The panel displays representative H&E-stained kidney sections (scale bar = 50 μm) for T2DM experimental groups. The images include untreated T2DM mice evaluated at 2, 8, and 24 h following i.p. injection of 2 (**A**), 5 (**B**), or 10 (**C**) mg/kg LPS, alongside T2DM mice challenged with 5 mg/kg LPS under three different DAPA regimens (Preventive (**D**), Therapeutic (**E**), and Whole-course (**F**)) at the corresponding time points. (Note: The corresponding semi-quantitative injury scores for these groups are presented in the panels (**A**–**F**) of Fig. 4). DAPA, dapagliflozin; h, hours; H&E, hematoxylin and eosin; i.p., intraperitoneal; LPS, lipopolysaccharide; T2DM, type 2 diabetes mellitus

Histopathological analysis provided independent structural validation of the functional data (Figs. 4, 5). In wild-type mice, endotoxemia resulted in dose- and time-dependent injury with evident self-repair at lower doses. In contrast, T2DM mice exhibited baseline chronic pathology. Following the LPS challenge, they sustained more severe acute damage and markedly impaired repair across all doses.

In T2DM mice subjected to moderate-dose LPS (5 mg/kg), the established model for timing intervention, DAPA treatment conferred distinct structural benefits. Semi-quantitative scoring revealed that at baseline, T2DM mice exhibited chronic injury. LPS challenge dramatically increased acute injury scores, which peaked at 8 h. All DAPA regimens attenuated this acute injury; however, the whole-course regimen showed the most pronounced effect, achieving near-normal acute scores by 24 h.

Notably, by 24 h, treated kidneys—especially in the therapeutic and whole-course groups—exhibited substantial architectural restoration, whereas Scr levels had not fully normalized (Fig. 3C). This observation suggests that tubular structural reconstruction may precede the full recovery of glomerular filtration function during the repair phase of AKI.

## Discussion

In the present study, we demonstrate that the therapeutic efficacy of the SGLT2 inhibitor DAPA in SA-AKI varies depending on intervention timing, particularly within the pathological context of T2DM. By employing continuous, real-time tGFR monitoring, we captured dynamic trajectories of renal decline and recovery that are often masked by conventional static biomarkers. Our observations reveal that while T2DM exacerbates the severity and impairs the repair of endotoxemia-induced AKI, both therapeutic (post-insult) and whole-course DAPA administration appear to provide superior renoprotection compared to preventive treatment alone. Notably, the therapeutic regimen significantly attenuated the decline in GFR as early as 2 h post-insult, suggesting a rapid effect that aligns with the acute pharmacodynamic action of SGLT2 inhibition. These results potentially broaden the traditional view of SGLT2 inhibitors from chronic metabolic stabilizers to acute-phase interventional agents that may target actively evolving renal injury in the diabetic-endotoxemic kidney.

This timing-dependent effect may help reconcile the discrepancy between the benefits of SGLT2 inhibitors in chronic kidney disease and the neutral findings reported in acute critical-care settings [12]. Unlike chronic diabetic nephropathy, which develops within a persistent maladaptive metabolic and hemodynamic milieu [15], SA-AKI evolves rapidly through a sequential injury cascade. Our data suggest that the optimal window for SGLT2 inhibition may coincide with the acute endotoxemic insult rather than the pre-challenge baseline. Therefore, the neutral outcomes of previous intensive care unit (ICU) pilot studies [12] may partly reflect heterogeneous intervention timing within this rapidly changing injury trajectory, rather than an inherent lack of drug efficacy.

The high-fat diet (HFD)/streptozotocin (STZ) model used in this study induces a mixed metabolic and β-cell injury phenotype that closely mirrors human T2DM but also carries distinct implications for SA-AKI susceptibility. Chronic HFD feeding establishes insulin resistance and lipotoxicity, while low-dose STZ causes partial β-cell dysfunction, together producing sustained hyperglycemia without complete β-cell ablation. This “two-hit” metabolic environment primes the kidney for heightened inflammatory and oxidative responses upon a secondary septic challenge. Although the precise molecular mechanisms are multifactorial, prior studies have identified several contributing pathways. Notably, the combination of hyperglycemia and elevated free fatty acids upregulates renal Gα12 expression, which activates the inositol-requiring enzyme 1 alpha (IRE1α)-activating transcription factor 6 (ATF6) arm of the endoplasmic reticulum stress pathway and subsequently triggers NLR family pyrin domain containing 3 (NLRP3) inflammasome-dependent pyroptosis [16]. Additionally, activation of necroptosis factor kappa B (NF-κB) signaling has been implicated in the exaggerated inflammatory response to LPS in this model [17]. Consequently, the HFD/STZ model establishes baseline lipid-induced tubular stress and a low-grade inflammatory state, which increases susceptibility to subsequent SA-AKI. This heightened baseline vulnerability provides a sensitive and translationally relevant background for evaluating the renoprotective efficacy of DAPA.

A possible hemodynamic explanation for this timing-dependent phenotype involves TGF, although this mechanism was not directly tested in the present study. SGLT2 inhibition is known to increase distal sodium delivery to the macula densa, which may activate TGF and induce afferent arteriolar vasoconstriction [15], a microvascular response previously confirmed in vivo using multiphoton microscopy by Kidokoro et al [18]. In the T2DM kidney, this TGF-mediated constriction has been proposed to alleviate maladaptive glomerular hyperfiltration and barotrauma [19]. During acute endotoxemia, regulation of glomerular vascular tone might also influence downstream microcirculatory perfusion. By dampening dysregulated hyperperfusion, DAPA could potentially facilitate redistribution of intrarenal blood flow toward the vulnerable peritubular capillary network and reduce ischemic injury. Consistent with this hemodynamic possibility, we observed slightly higher baseline Scr levels in WT mice receiving DAPA than in the control group. Although Scr levels were higher than those in the control group, they remained within the normal reference range. This pattern is consistent with the KDIGO 2024 Clinical Practice Guideline, which describes the early mild increase in serum creatinine associated with SGLT2 inhibitors as a benign and reversible hemodynamic effect indicative of target engagement, rather than acute nephrotoxicity [20]. In our timing-intervention model, the therapeutic regimen administered 0.5 h after LPS challenge produced greater functional protection than preventive exposure alone. Specifically, at 8 h post-LPS challenge, the therapeutic group maintained significantly lower Scr levels and superior GFR recovery compared with the preventive group. This temporal pattern is compatible with the possibility that the acute pharmacodynamic actions of SGLT2 inhibition, potentially including but not limited to hemodynamic modulation, may be more beneficial when engaged during the evolving endotoxemic injury phase.

Beyond these hemodynamic considerations, the superior functional rescue observed in the therapeutic group also raises the possibility that metabolic adaptation may contribute to the observed phenotype. In the hyperinflammatory environment of SA-AKI, renal tubular cells often undergo a maladaptive metabolic shift—reminiscent of the Warburg effect—where oxidative phosphorylation is suppressed in favor of less efficient glycolysis [21]. Because the therapeutic regimen provided greater functional rescue than preventive administration, we speculate that timely DAPA intervention may help attenuate this acute metabolic stress. This interpretation is compatible with mechanistic evidence showing that SGLT2 inhibition can suppress hypoxia-inducible factor-1 alpha (HIF-1α)-mediated metabolic switching, preserve mitochondrial fatty acid oxidation (FAO), and mitigate energetic failure in the diabetic kidney [22]. However, because we did not directly measure HIF-1 α signaling, FAO, or mitochondrial respiration, this explanation remains hypothesis-generating.

Crucially, as proposed by Packer, SGLT2 inhibitors may induce a transcriptional state resembling nutrient deprivation by counteracting nutrient-excess signaling [23]. This fasting-like state may activate key energy sensors, including adenosine monophosphate-activated protein kinase (AMPK) and sirtuin 1 (SIRT1), which are involved in cellular metabolic homeostasis. In the context of acute inflammation, DAPA-mediated AMPK activation has been proposed to promote the utilization of ketone bodies, providing an alternative energy substrate that bypasses impaired glycolysis. Furthermore, upregulation of AMPK/SIRT1 signaling may enhance autophagic flux, facilitating the clearance of damaged mitochondria and reducing the release of mitochondrial damage-associated molecular patterns (DAMPs). These processes could contribute to reduced intracellular oxidative stress and secondary inflammatory signaling, potentially explaining the lower levels of pro-inflammatory cytokines such as tumor necrosis factor-alpha (TNF-α), interleukin-6 (IL-6), and interleukin-1 beta (IL-1β) reported in studies of SGLT2 inhibition [23]. In the present study, however, these pathways were inferred from prior literature rather than directly measured.

Mitochondrial preservation may represent another possible mechanism linking metabolic adaptation to the renal functional protection observed in our model. In the T2DM kidney, mitochondria are chronically primed by persistent hyperglycemia and lipotoxicity, leading to excessive reactive oxygen species (ROS) production and impaired antioxidant defenses even before the onset of sepsis [24]. Acute LPS challenge may further amplify this pre-existing oxidative stress and promote mitochondrial dysfunction. By reducing active tubular transport workload, SGLT2 inhibition has been reported to lower tubular oxygen consumption (QO_2_) [25], which could alleviate the hypoxic burden on the mitochondrial electron transport chain and limit superoxide generation. In parallel, preservation of mitochondrial FAO pathways [22] and potential activation of the nicotinamide adenine dinucleotide (NAD⁺)/SIRT1 signaling axis [26] may contribute to mitochondrial quality control and biogenesis. This putative mitochondrial stabilization could also be linked to inflammatory modulation: the SGLT2 inhibition-associated shift toward ketone body production, particularly β-hydroxybutyrate, together with reduced mitochondrial ROS, has been shown to suppress activation of the NLRP3 inflammasome [27]. Such metabolic-inflammatory crosstalk may reduce IL-1β maturation and blunt downstream cytokine amplification involving TNF-α and IL-6. Although these mechanisms provide a biologically plausible framework for interpreting our rapid tGFR recovery phenotype captured by real-time transcutaneous GFR monitoring [28], direct validation using mitochondrial, metabolic, and inflammatory assays will be required.

Consistent with this possible metabolic preservation, our 24-h histological observations highlighted a temporal dissociation in which substantial tubular architectural repair appeared to precede full normalization of conventional static biomarkers. We hypothesize that by reducing the acute cellular workload, DAPA may spare the energy required to support structural rebuilding, thereby providing a structural prerequisite for subsequent physiological recovery. Furthermore, this apparent delay in biomarker normalization may partially reflect the inherent kinetic lag of traditional circulating biomarkers, such as Scr and BUN. In this context, the advantage of real-time transcutaneous GFR monitoring becomes evident. Unlike conventional biomarkers that often lag behind actual changes in renal function because of delayed systemic clearance, tGFR sensitively captured the immediate and continuous functional recovery trajectory. This suggests that the early energy-sparing protection conferred by DAPA may facilitate structural healing, which then translates into the timely physiological rescue observed in our dynamic functional profiles. Nevertheless, the causal relationship between reduced workload, histological repair, and functional recovery remains to be established.

Current ADA guidelines recommend SGLT2 inhibitors for many patients with T2DM, particularly those with CKD, heart failure, or elevated cardiorenal risk; therefore, a substantial proportion of diabetic patients may already be exposed to these agents when sepsis develops [5]. This clinical context supports the relevance of our preventive experimental arm. However, our dynamic tGFR profiles showed that DAPA administration during the acute insult, either as a whole-course or therapeutic regimen, provided greater preservation of renal function and tubular architecture than preventive exposure alone. These findings identify intervention timing as a potentially important determinant of SGLT2 inhibitor-associated renoprotection in diabetic SA-AKI. Together, these findings support the concept that continuation or timely initiation of SGLT2 inhibitor therapy during acute septic episodes may offer renal protective benefits in diabetic patients who develop SA-AKI, while providing a preclinical rationale for future clinical studies to determine its efficacy, safety, and optimal timing before clinical translation.

### Limitations

Several limitations should be acknowledged. First, this study focused primarily on functional and histological outcomes. Although our findings demonstrate a clear timing-dependent efficacy of DAPA, we did not directly assess inflammatory cytokines, oxidative stress, mitochondrial function, or other downstream molecular pathways. Because SGLT2 inhibitors are known to induce metabolic reprogramming, the lack of transcriptomic, metabolomic, or fluxomic analyses limits our ability to define the precise molecular drivers underlying the stronger protection observed in the whole-course and therapeutic regimens.

Second, intraperitoneal LPS injection induces sterile endotoxemia rather than polymicrobial sepsis. Nevertheless, recent organism-wide transcriptomic analyses have shown substantial overlap in multi-tissue gene expression profiles between LPS-induced endotoxemia and the gold-standard cecal ligation and puncture model, particularly during the early acute phase within 24 hours [14]. Thus, the graded LPS model used here provides a reproducible platform for studying early endotoxemia-associated AKI, while not fully recapitulating the complexity of clinical SA-AKI.

Third, the 0.5-h therapeutic window represents early interception rather than rescue of established severe AKI. Our tGFR data showed a marked functional decline as early as 2 h after LPS challenge, supporting the rationale for early intervention in this rapidly evolving model. However, this timing may not reflect delayed clinical presentation in the ICU, where sepsis is often recognized hours or days after the initial insult. Whether DAPA can restore renal function when administered at later stages remains to be determined.

Fourth, our T2DM model was induced by HFD combined with low-dose STZ, which generates a mixed metabolic and β-cell cytotoxic phenotype. This model is widely used and recapitulates key features of human T2DM, but it does not fully isolate the effects of chronic hyperglycemia from those of lipotoxicity or STZ-related direct tubular injury. Alternative T2DM models, such as genetically obese db/db or ob/ob mice, which develop hyperglycemia on a background of leptin signaling deficiency without chemical β-cell damage, or diet-induced obesity models that lack STZ, might exhibit different baseline renal inflammatory profiles and distinct susceptibility to SA-AKI [29, 30]. It remains possible that the renoprotective efficacy of DAPA might differ across models, potentially depending on the relative contributions of hyperglycemia, insulin resistance, and pre-existing tubular stress. Thus, while our findings provide strong evidence for timing-dependent protection in the HFD/STZ model, future studies using complementary T2DM models are needed to determine the generalizability of the observed timing-dependent effects.

Fifth, these findings were obtained in an animal model and therefore require cautious interpretation before clinical translation. Future clinical studies are needed to determine whether continuation or timely initiation of SGLT2 inhibitor therapy during acute septic episodes can improve renal outcomes in diabetic patients with SA-AKI. Such studies should also carefully define the optimal timing, therapeutic window, and safety profile of this strategy in the setting of acute sepsis.

## Conclusions

This study demonstrates that T2DM exacerbates endotoxemia-associated AKI and impairs renal repair capacity. The renoprotective efficacy of the SGLT2 inhibitor DAPA in this setting is greatly influenced by the timing of intervention, with treatment initiated after the endotoxemic insult or maintained throughout the entire course proving superior to preventive administration alone in preserving renal function, attenuating tubular injury, and promoting histological repair. Translating these experimental insights into clinical implications, our findings suggest that for patients with underlying T2DM, the renoprotective benefits of SGLT2 inhibition are maximized when therapy is actively continued or promptly initiated upon the onset of clinical sepsis. Our results provide a preclinical rationale for the prompt initiation or continuation of SGLT2 inhibitor therapy in diabetic patients at risk of, or presenting with SA-AKI, and highlight the need for future clinical studies to validate these timing-dependent effects.

## Methods

### Ethical statement and animal preparation

All animal procedures were approved by the Institutional Animal Care and Use Committee of the First Affiliated Hospital of Harbin Medical University (Approval No. IACUC-2023022). This study was designed, performed, and reported in strict accordance with the ARRIVE 2.0 guidelines and the “3Rs” principles. Male C57BL/6 mice (5 weeks old) were housed under standard specific pathogen-free conditions with ad libitum access to food and water. Detailed laboratory information, extended husbandry conditions, and humane endpoint criteria are provided in Supplementary Material, Section S1.

### Experimental design and grouping

This study employed a factorial design to address two primary aims: first, to evaluate the impact of pre-existing T2DM on the severity and progression of endotoxemia-associated AKI; and second, to determine whether the renoprotective efficacy of DAPA depends on the timing of administration relative to the acute endotoxemic insult. Because the chronic cardiorenal benefits of SGLT2 inhibitors in stable T2DM are already well-established clinically [10, 31, 32], we specifically focused on the dynamically evolving acute phase; thus, to align with clinical relevance, a stable T2DM control group receiving DAPA without LPS was not included.

**Fig. 6:**
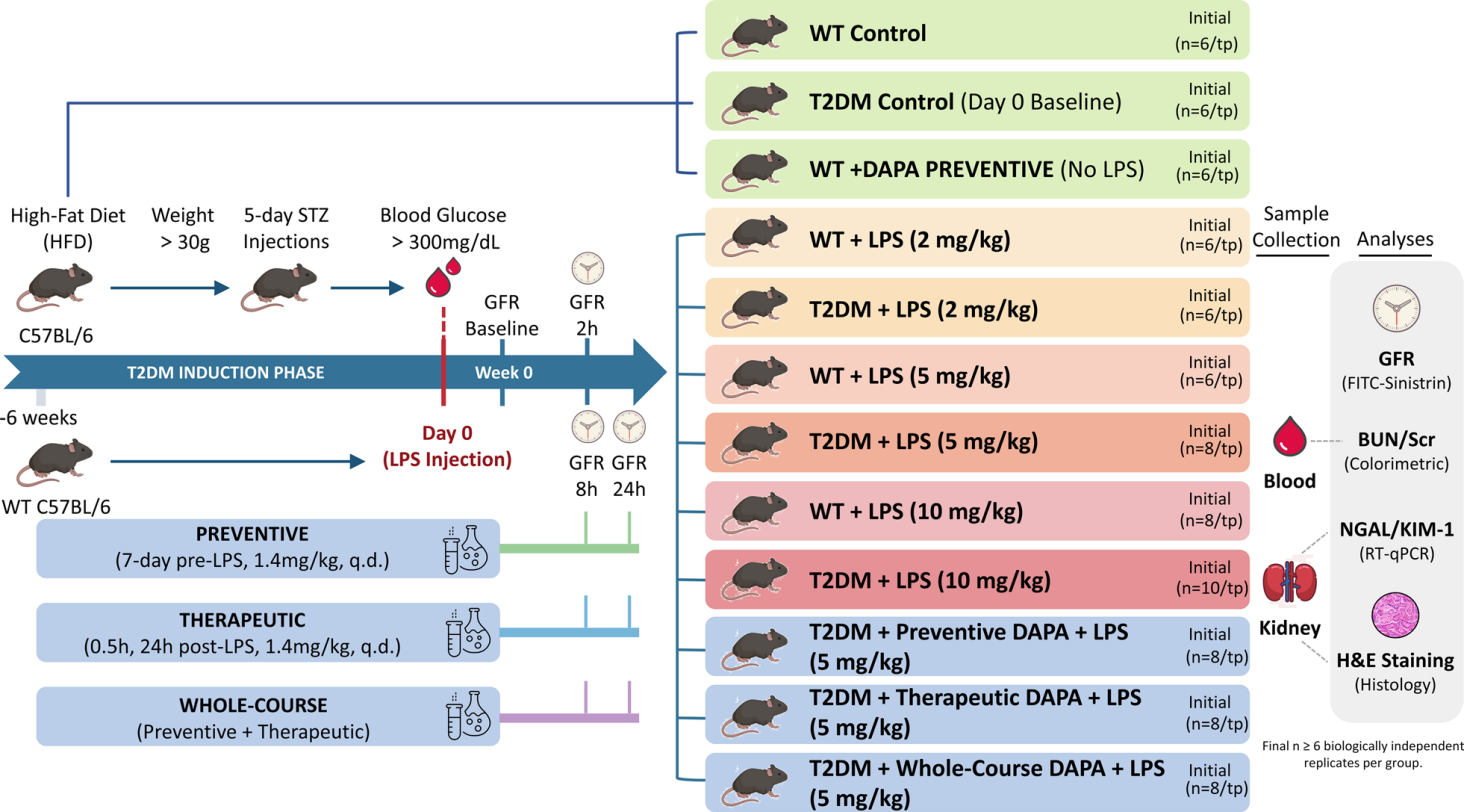
Study design, LPS-induced injury timeline, and schematic of dapagliflozin administration regimens. Male C57BL/6 mice (WT and T2DM) received LPS (0, 2, 5, or 10 mg/kg, i.p.). To evaluate the efficacy of different administration timings, three DAPA regimens (Preventive, Therapeutic, and Whole-course; 1.4 mg/kg/day, p.o.) were applied to T2DM mice challenged with a moderate LPS dose (5 mg/kg), totaling 12 experimental groups. Mice were assessed at 2, 8, and 24 h post-LPS (0 mg/kg as baseline). To account for anticipated dose-dependent mortality, initial group sizes were scaled to 6, 8, or 10 animals per time point (tp). Terminal measurements included tGFR, followed by blood and kidney collection for BUN/Scr, *NGAL/KIM-1* mRNA, and H&E staining. Animals that died prematurely or showed abnormal baseline renal function were excluded, ensuring all reported groups retained a final sample size of at least 6 biologically independent replicates per group. WT, wild-type; T2DM, type 2 diabetes mellitus; LPS, lipopolysaccharide; i.p., intraperitoneal; DAPA, dapagliflozin; p.o., per os (oral administration); h, hours; tp, time point; tGFR, transcutaneous glomerular filtration rate; BUN, blood urea nitrogen; Scr, serum creatinine; NGAL, neutrophil gelatinase-associated lipocalin; KIM-1, kidney injury molecule-1; H&E, hematoxylin and eosin

To address these aims, mice were randomized into 12 experimental groups (Fig. 6) to compare baseline differences between WT and T2DM mice, time- and dose-dependent responses to varying severities of LPS-induced AKI evaluated at 2, 8, and 24 h post-injection, and the efficacy of three DAPA timing regimens in T2DM mice challenged with a moderate LPS dose. The selection of this moderate LPS dose was determined based on existing research [14] and preliminary dose-titration pilot studies. These pilot experiments demonstrated that this challenge reliably induces a robust, sub-lethal AKI, thereby providing an adequate 24-h therapeutic window to evaluate pharmacological efficacy while avoiding the confounding extremes of insufficient injury at lower doses or premature fatal shock at higher doses.

To minimize bias and adhere to the ARRIVE guidelines, the daily order of experimental procedures was randomized, and outcome assessments (such as histopathological scoring) were conducted by investigators blinded to the group assignments. Due to the large total number of animals required, mice from multiple housing batches were utilized, with preliminary analyses confirming no significant batch-to-batch variations.

Based on pilot mortality data, a target final sample size of six animals per group was established to ensure adequate statistical power. To account for dose-dependent mortality, initial group sizes were adjusted according to disease status and LPS dose severity. In WT control mice, six animals per group per time point were used for low- and moderate-dose LPS challenges, and eight were used for high-dose challenge. In T2DM mice, six, eight, and ten animals per group per time point were used for low-, moderate-, and high-dose LPS challenges, respectively. The DAPA intervention groups were based on the T2DM moderate-dose LPS model and therefore included eight animals per group per time point. Animals dying prematurely or exhibiting marked baseline renal abnormalities were excluded, ensuring that all reported groups retained at least six biologically independent replicates.

### Induction of type 2 diabetes by high-fat diet and streptozotocin

T2DM was induced by combining a HFD with low-dose STZ injections. This well-established model recapitulates the insulin resistance and progressive β-cell dysfunction characteristic of human T2DM [33]. Mice were maintained on an HFD throughout the study. After 4–6 weeks of HFD feeding, when individual body weight exceeded 30 g (indicative of developed insulin resistance), diabetes was initiated by intraperitoneal injections of STZ (35 mg/kg/day) for 5 consecutive days. The STZ dosage was determined based on established literature and optimized through preliminary pilot experiments. This low-dose regimen, combined with an HFD, reliably induced stable hyperglycemia while avoiding severe acute beta-cell toxicity. To minimize acute metabolic fluctuations, mice were fasted for 5 h before the first STZ injection and for 1.5 h after the injection, but had free access to food at all other times [33–35]. Diabetes was confirmed by measuring random blood glucose levels; mice with consecutive readings > 300 mg/dL were included in the T2DM group (details in Supplementary Material, Section S2) [36].

### LPS-induced endotoxemia model as a surrogate for clinical sepsis

An experimental endotoxemia model, serving as a well-established surrogate for early-phase sepsis [14], was induced by the intraperitoneal injection of LPS. Although LPS primarily elicits sterile endotoxemia rather than the full complexity of polymicrobial clinical sepsis, recent comprehensive transcriptomic analyses have demonstrated a high degree of similarity in organism-wide multi-tissue responses between LPS-induced endotoxemia and the gold-standard CLP (cecal ligation and puncture) sepsis model, particularly during the acute phase (up to 24 h) [14]. Therefore, to comprehensively model an endotoxemia severity gradient, mice received LPS at low (2 mg/kg), moderate (5 mg/kg), or high (10 mg/kg) doses. This graded approach closely parallels the molecular trajectories observed in varying severities of clinical polymicrobial sepsis [14]. Crucially, this spectrum allowed us to systematically characterize the distinct phenotypic vulnerabilities of T2DM mice across specific clinical scenarios: the low dose models mild sepsis, the moderate dose mirrors moderate-to-severe sepsis with established organ dysfunction, and the high dose represents fulminant septic shock. Based on this robust phenotypic mapping, the moderate dose was purposefully selected for evaluating the DAPA timing regimens, as it provides an ideal sub-lethal 24-h therapeutic window with a wide dynamic range to accurately track functional and structural pharmacological rescue. The successful establishment of systemic inflammation was confirmed by the observation of typical clinical signs, including fever, tachycardia, tachypnea, lethargy, piloerection, reduced activity, and ocular discharge [37].

### Timing of dapagliflozin administration

Dapagliflozin was administered orally at a dose of 1.4 mg/kg/day according to three timing regimens. This dosage was mathematically derived through interspecies allometric scaling based on foundational pharmacokinetic data [38]. To prevent potential toxicity in our obese T2DM model, a Lean Body Mass (LBM)-based dosing strategy was implemented. The absolute dose was capped based on an ideal lean weight of 30 g to ensure consistent and safe target organ exposure within the human-equivalent therapeutic window [28].

In the preventive regimen, it was given daily for 7 consecutive days before the LPS challenge. In the therapeutic regimen, a first dose was administered at 0.5 h post-LPS. This 0.5-h post-LPS window was strategically selected based on established proof-of-concept protocols for SGLT2 inhibitors in diabetic endotoxemia, as well as the pharmacokinetics of DAPA in rodents [28, 39]. As the oral peak plasma concentration (Tmax) typically occurs at 1–2 h post-gavage, this timing ensures that maximal systemic and renal drug exposure synchronizes with the peak of the acute LPS-induced inflammatory and hemodynamic crisis (typically 1–3 h post-insult). For mice observed until the 24-h endpoint, a second dose was administered at 24 h post-LPS. Although the elimination half-life of DAPA in mice is short, a strict 24-h (once-daily, q.d.) interval was maintained to adhere to standard preclinical protocols [28, 39], account for the prolonged pharmacodynamic (PD) effects of the drug, and, importantly, minimize additional stress from repeated oral gavage in critically ill endotoxemic mice. This design allows the distinction between a single early intervention and maintained drug action. The whole-course regimen combined both the preventive and therapeutic schedules. This multi-window framework also accounts for the chronopharmacological sensitivity of SGLT2 inhibitors in mice, as light-phase administration is associated with maximal efficacy [39]. The dose and timing framework was based on established pharmacological data (detailed justifications provided in Supplementary Material, Section S3) [28, 38].

### Transcutaneous glomerular filtration rate measurement

Real-time GFR was measured non-invasively via transcutaneous detection of fluorescein isothiocyanate (FITC) -sinistrin clearance after tail vein injection [40]. GFR measurements were made at baseline and 2, 8, and 24 h post-LPS, with analysis using a three-compartment model (MBStudio v.22) (Detailed instrumentation, tracer preparation, and measurement procedures are described in Supplementary Material, Section S4).

### Biochemical, molecular, and histological analyses

Blood and kidney tissue samples were collected at designated time points. BUN and Scr were measured using standard automated and colorimetric assays, respectively (details in Supplementary Material, Section S7). Total RNA from kidney tissue was reverse-transcribed, and mRNA expression of NGAL and KIM-1 was quantified by real-time quantitative polymerase chain reaction (RT-qPCR) (SYBR Green method, normalized to GAPDH) (Primer sequences are provided in Supplementary Material, Section S5 and Table S1). For histopathology, kidney sections were stained with hematoxylin and eosin (H&E) and evaluated by a blinded observer using a semi-quantitative scoring system (details in Supplementary Material, Section S6 and Table S2).

### Statistical analysis

Data are expressed as mean ± SEM. Normality was assessed using the Shapiro–Wilk test, and all quantitative datasets met the assumptions for parametric analysis. To preserve statistical power and avoid the penalty of excessive multiple testing, an all-to-all statistical comparison across the 12 experimental groups was not performed. Instead, comparisons were prospectively restricted to the pre-defined analytical subsets aligned with the primary study aims described in Sect. "Experimental Design and Grouping". For comparisons between different groups at a single time point, statistical analyses were performed using two-way analysis of variance (ANOVA) with Tukey’s post hoc test. For the repeated measures analysis of longitudinal datasets (specifically, the GFR time-series data measured consecutively at baseline, 2, 8, and 24 h within the same animals), Linear Mixed-Effects Models were employed to account for the within-subject correlation over time. A *P* value < 0.05 was considered statistically significant. All analyses were executed using GraphPad Prism 10.4.0.

## Supplementary Information

Additional file 1.

Additional file 2.

Additional file 3.

## Data Availability

All data generated or analyzed during this study are included in this published article and the supplementary material.

## Abbreviations

ADA: American diabetes association
AKI: Acute kidney injury
AMPK: Adenosine monophosphate-activated protein kinase
ANOVA: Analysis of variance
ATF6: Activating transcription factor 6
BUN: Blood urea nitrogen
CLP: Cecal ligation and puncture
DAMPs: Damage-associated molecular patterns
DAPA: Dapagliflozin
FAO: Fatty acid oxidation
FITC: Fluorescein isothiocyanate
GAPDH: Glyceraldehyde-3-phosphate dehydrogenase
GFR: Glomerular filtration rate
H&E: Hematoxylin and eosin
HFD: High-fat diet
HIF-1α: Hypoxia-inducible factor-1 alpha
ICU: Intensive care unit
IL-1β: Interleukin-1 beta
IL-6: Interleukin-6
IRE1α: Inositol-requiring enzyme 1 alpha
KIM-1: Kidney injury molecule-1
LPS: Lipopolysaccharides
mRNA: Messenger ribonucleic acid
NAD⁺: Nicotinamide adenine dinucleotide
NF-κB: Necroptosis factor kappa B
NGAL: Neutrophil gelatinase-associated lipocalin
NLRP3: NLR family pyrin domain containing 3
QO_2_: Tubular oxygen consumption
ROS: Reactive oxygen species
RT-qPCR: Real-time quantitative polymerase chain reaction
RNA: Ribonucleic acid
SA-AKI: Sepsis‑associated acute kidney injury
Scr: Serum creatinine
SGLT2: Sodium–glucose cotransporter 2
SIRT1: Sirtuin 1
STZ: Streptozotocin
T2DM: Type 2 diabetes mellitus
TGF: Tubuloglomerular feedback
tGFR: Transcutaneous glomerular filtration rate
TNF-α: Tumor necrosis factor-alpha
WT: Wild-type
