# Supplementary material for: Timing-dependent renal protection of dapagliflozin in endotoxemic diabetic mice by real-time GFR and biomarkers
Source: Intensive Care Med Exp. 2026 Jul 1;14:86. doi: 10.1186/s40635-026-00940-2 (PMC13323439; doi:10.1186/s40635-026-00940-2)
Supplement: Supplementary file 1 — Additional file 1. [file 40635_2026_940_MOESM1_ESM.docx]

**Supplementary Methods**

**S1. Animal Housing and Ethics**

Housing Conditions: Animals were housed in a sterile, individually ventilated cage (IVC) system. The environment was maintained at a constant temperature of 23°C and a relative humidity of 60% ± 10%, under a 12-hour light/12-hour dark cycle. Bedding, feed, and drinking water were autoclaved. Bedding was changed twice a week.

Environmental enrichment was provided in the form of nesting material and chewable objects to promote species-typical behaviors and well-being. To alleviate potential pain or distress, animals were closely monitored after invasive procedures (e.g., intraperitoneal injections). For prolonged procedures such as transcutaneous GFR monitoring, anesthesia was maintained with isoflurane to ensure freedom from pain. Soft food and water were readily available throughout the study period if needed. Humane endpoints were predefined and included the following criteria: inability to access food or water autonomously, severe dyspnea, profound weakness (inability to stand), hypothermia (<32°C), or body weight loss exceeding 20% of the initial weight. Animal health and behavior were monitored at least twice daily by trained personnel.

Any animal reaching a humane endpoint was euthanized immediately. The predetermined observational time points (2, 8, 24 h) were not used as humane endpoints.

**Ethical Statement**

All animal procedures were strictly performed in accordance with the protocol approved by the Animal Management and Welfare Committee of the First Affiliated Hospital of Harbin Medical University (Approval No. IACUC-2023022).

**S2. Type 2 Diabetes Mellitus (T2DM) Model Induction**

**High-Fat Diet (HFD):** Mice were fed an HFD (Synergy Bio, product code: XTHF60) with a caloric composition of 60% fat, 20% carbohydrate, and 20% protein.

**Streptozotocin (STZ) Preparation and Injection:** STZ was purchased from Abisin (Cat. No.: abs812888-1g). It was freshly dissolved in pre- cooled 0.1 M sodium citrate buffer (*p*H 4.5) immediately before injection. Based on classical protocols and our preliminary optimizations, STZ was administered intraperitoneally at a daily dose of 35 mg/kg for 5 consecutive days. Mice were fasted for 5 hours before the first STZ injection and then returned to the HFD. In our main experimental cohort, this standardized regimen yielded a high diabetes induction success rate of approximately 80%, with minimal acute toxicity prior to the subsequent experimental procedures.

**Confirmation of Diabetes:** Diabetes was confirmed by meeting both of the following criteria: (1) two consecutive fasting blood glucose measurements > 300 mg/dL; and (2) an oral glucose tolerance test showing a significantly increased area under the curve and delayed glucose clearance. This criterion is used in both the main text and supplementary materials.

**S3. Drug Preparation and Administration**

**Dapagliflozin:** The drug was purchased from Sigma-Aldrich (Cat. No.: SML2804-25MG).

**Detailed Preparation Protocol:** Dapagliflozin powder was accurately weighed and first dissolved in dimethyl sulfoxide (DMSO, Abisin, abs9187-100ml) to prepare a 2 mg/mL stock solution. This stock was diluted to the target concentration with a 1% aqueous solution of carboxymethylcellulose sodium (CMC-Na) immediately before administration. The final concentration of DMSO in the gavage solution was less than 1% (v/v).

**Vehicle Control:** Control animals received an equal volume of the 1% CMC-Na solution containing the same proportion of DMSO.

**Justification for Selecting Dapagliflozin (DAPA) over other SGLT2 Inhibitors:**

The selection of DAPA was driven by three primary methodological considerations:
(1) Methodological Continuity: To rigorously investigate the “intervention timing” hypothesis, we maintained methodological continuity with foundational studies (Chi et al., 2022) demonstrating DAPA’s efficacy in alleviating LPS-induced AKI in T2DM mice. Using the same agent avoids uncontrolled confounding variables related to the inherent molecular differences of other SGLT2 inhibitors.
(2) Pharmacokinetic Stability in Sepsis: Severe endotoxemia induces acute hepatic stress. Because DAPA is predominantly metabolized via the UGT1A9 enzyme, it maintains stable systemic exposure without requiring dose adjustments during mild-to-moderate hepatic impairment, thereby minimizing secondary confounding effects from hepato-renal crosstalk. Furthermore, agents with partial SGLT1 inhibition (e.g., canagliflozin) were strictly excluded to prevent the exacerbation of sepsis-induced gastrointestinal barrier dysfunction.
(3) Alignment with Metabolic Mechanisms: DAPA is the pioneering pharmacological probe used in foundational studies demonstrating renal medullary metabolic offloading and the reduction of oxygen consumption, which perfectly aligns with our tested bioenergetic hypothesis.

Dapagliflozin: The drug was purchased from Sigma-Aldrich (Cat. No.: SML2804-25MG).

**Justification for Dose Selection and Calculation:**

The dosage of 1.4 mg/kg/day for dapagliflozin (DAPA) was not chosen arbitrarily; rather, it was mathematically derived through allometric scaling and intentionally adjusted for the obese phenotype of our T2DM model:

(1) Allometric Scaling from Preclinical PK Data: Based on the foundational preclinical pharmacokinetic study by Obermeier et al.^1^, a dose of 1.0 mg/kg provides robust systemic exposure and target engagement in rats. The precise dosage for our mice was derived using the standard interspecies dose equivalence method. The calculation yielded a murine dose of 1.4 mg/kg (Calculated as: 1.0 mg/kg×0.2 kg×(0.14/0.02 kg)=1.4 mg/kg).

(2) Validation via Human Equivalent Dose (HED): The standard clinical therapeutic dose of DAPA is 5–10 mg/day for a 60 kg adult (i.e., 0.083–0.167 mg/kg). Using the standard HED conversion factor for mice (12.3), the expected equivalent murine dose ranges from approximately 1.02 to 2.05 mg/kg. Our calculated dose of 1.4 mg/kg sits optimally within this safe and established therapeutic window, consistent with prior literature on murine T2DM models^2^.

(3) Lean Body Mass (LBM)-Based Dosing Strategy: To strictly avoid overdosing in our obese T2DM model, an LBM-based dosing strategy was implemented. Although the high-fat diet (HFD)-fed T2DM mice exhibited significant weight gain (reaching 40–45 g), the absolute dose per animal was capped based on an ideal lean weight of 30 g (yielding an absolute drug dose of 0.042 mg per mouse). Because SGLT2 inhibitors do not significantly distribute into adipose tissues, dosing based on total body weight would lead to disproportionately high acute plasma concentrations. This LBM strategy ensures consistent and safe therapeutic exposure at the target organ, minimizing toxicity during the severe systemic endotoxemic challenge.

**Pharmacological and Pharmacokinetic Justification for Dosing Timing:**

The specific timing framework for dapagliflozin (DAPA) administration was strategically designed based on its well-established pharmacokinetics (PK) and chronopharmacology in rodents:

1. Alignment of Drug Peak with Injury Peak (The +0.5h Rationale): Following oral administration in mice, DAPA is rapidly absorbed, reaching its peak plasma concentration (Tmax) within approximately 1 to 2 hours^1^. In our model, the intraperitoneal injection of LPS induces an acute inflammatory cascade and renal hemodynamic collapse that typically peaks between 1 and 3 hours post-insult. Administering the therapeutic dose of DAPA at 0.5 hours post-LPS allows the maximal systemic and renal drug exposure to precisely synchronize with the peak of the acute injury phase, modeling a prompt and targeted clinical interception.
2. Chronopharmacological Efficacy: Recent evidence demonstrates that DAPA exhibits pronounced chronopharmacology in C57BL/6J mice. Yoshioka et al. (2019) reported that oral DAPA administered during the light phase (ZT2) significantly improved glycemic control and attenuated adipocyte inflammation^3^, whereas administration during the dark phase (ZT14) yielded minimal to no effects. Because our experimental interventions were consistently conducted during the light phase, the +0.5h regimen optimally aligns with the physiological window of maximal pharmacological sensitivity for this species.
3. Maintenance of Pharmacological Coverage (The 24h Interval): Although the elimination half-life (T1/2) of DAPA in mice (approximately 4–6 hours) is shorter than in humans, the second dose at 24 hours was administered strictly to adhere to the standardized clinical once-daily (q.d.) regimen. A 24-hour interval was utilized rather than a shorter, PK-matched interval (e.g., every 8 hours) based on three critical scientific and practical factors:

Pharmacodynamic (PD) Persistence: SGLT2 inhibitors exhibit prolonged downstream metabolic and hemodynamic reprogramming (e.g., tubuloglomerular feedback restoration and reduction of intraglomerular pressure) that persists well beyond systemic drug clearance, rendering the 24-hour interval sufficient to maintain pharmacological efficacy.

Preclinical Consensus: The once-daily oral administration of DAPA is the universally established standard in murine models of diabetes and AKI^2,3^. Adhering to this clinical q.d. analog ensures our outcomes are directly comparable to the existing body of literature.

**Minimization of Lethal Stress:** Repeated oral gavage (e.g., every 6–8 hours) induces profound mechanical stress and sympathetic activation. In mice already experiencing the severe systemic inflammatory storm of LPS-induced endotoxemia, such frequent handling would drastically exacerbate mortality and severely confound the inflammatory readouts.

Therefore, while the high lethality of the acute T2DM-endotoxemia model intrinsically limits the primary functional observation window to 24 hours, adhering to this strict q.d. protocol prevents methodological isolation. It ensures our experimental framework mimics continuous clinical care and remains robustly comparable to established preclinical literature, even though the biochemical readouts exactly at the 24-hour mark are predominantly driven by the preceding acute-phase dose.

**S4. Technical Details of Transcutaneous Glomerular Filtration Rate (GFR) Monitoring**

The monitoring of transcutaneous glomerular filtration rate was performed using a specialized transcutaneous fluorescence monitoring system (MediBeacon GmbH, Mannheim, Germany). To prevent signal scattering, the abdominal hair of the mice was removed 24 hours prior to the measurement. During the monitoring setup, the mice were briefly anesthetized with 1.5% to 2% isoflurane, and an optical sensor was securely fixed over the renal region using a double-sided adhesive patch. To ensure signal quality, the device was activated for 1 to 2 minutes prior to injection to record the tissue background autofluorescence. The FITC-sinistrin fluorescent tracer, serving as a specific marker for glomerular filtration, was prepared according to the manufacturer’s instructions and rapidly injected via the tail vein at a dose of 0.7 mg/kg. Following the injection, the mice regained consciousness, and the fluorescence decay curve was recorded continuously for 60 minutes in the conscious, freely moving animals.

Data analysis was conducted using the device’s dedicated MBStudio analysis software (version 22). Based on the validated methodology by Schreiber et al. (2012)^4^, the transcutaneous GFR calculation is independent of absolute fluorescence intensity and is instead derived from the elimination half-life (t1/2) of the tracer. Initially, the software calculates the GFR by fitting the data to a three-compartment kinetic model. Because conscious mice exhibit movement that commonly causes pressure-related motion artifacts, particularly during the early post-anesthesia period, specific strategies were applied to handle these artifacts. To mathematically eliminate such interference, the software utilizes a one-compartment kinetic model where the exponential fitting is strictly applied to the stable, single-exponential excretion phase, thereby deliberately bypassing the initial 10 to 15-minute distribution phase and early motion artifacts.

To ensure that the data extraction remained objective and blinded to operator bias, the entire fitting process and GFR calculation were executed automatically by the software algorithm. Finally, the resulting GFR values were intrinsically normalized to body weight utilizing a mouse-specific empirical conversion factor integrated within the software, yielding the final results expressed as mL/min/100g body weight.

**S5. Molecular Biology Analysis**

RNA Extraction and qPCR: Total RNA was extracted from kidney tissue using TRIzol reagent (Invitrogen). Reverse transcription was performed using the High-Capacity cDNA Reverse Transcription Kit (Applied Biosystems). Quantitative PCR was performed using the UltraSpeed SYBR Green PCR Master Mix (Bimake, B21203) on a QuantStudio series real- time PCR system.

Primer Sequences: The primer sequences used for qPCR are listed in the table below.

**S6. Renal Histopathological Scoring Criteria**

Renal injury was evaluated by a researcher blinded to the group allocation using a semi-quantitative scoring system under light microscopy. The detailed scoring criteria for acute and chronic injuries are described in the table below.

**S7. Other Assay Methods**

Blood Urea Nitrogen (BUN) and Serum Creatinine (Scr): Serum BUN was measured using an IDEXX Catalyst One automated biochemistry analyzer. Scr was determined using a commercial creatinine assay kit (microplate method, Nanjing Jiancheng Bioengineering Institute) strictly following the manufacturer's instructions.

Tissue Processing: Kidney tissues were fixed in 4% paraformaldehyde, routinely embedded in paraffin, sectioned at 4 μm thickness, and stained with Hematoxylin and Eosin (H&E).

**Supplementary References**

1. Obermeier M, Yao M, Khanna A, et al. In vitro characterization and pharmacokinetics of dapagliflozin (BMS-512148), a potent sodium-glucose cotransporter type II inhibitor, in animals and humans. *Drug Metab Dispos*. Mar 2010;38(3):405-14. doi:10.1124/dmd.109.029165

2. Chi PJ, Lee CJ, Hsieh YJ, Lu CW, Hsu BG. Dapagliflozin Ameliorates Lipopolysaccharide Related Acute Kidney Injury in Mice with Streptozotocin-induced Diabetes Mellitus. *Int J Med Sci*. 2022;19(4):729-739. doi:10.7150/ijms.69031

3. Yoshioka H, Ohishi R, Hirose Y, et al. Chronopharmacology of dapagliflozin-induced antihyperglycemic effects in C57BL/6J mice. *Obes Res Clin Pract*. Sep-Oct 2019;13(5):505-510. doi:10.1016/j.orcp.2019.08.001

4. Schreiber A, Shulhevich Y, Geraci S, et al. Transcutaneous measurement of renal function in conscious mice. *Am J Physiol Renal Physiol*. Sep 2012;303(5):F783-8. doi:10.1152/ajprenal.00279.2012
